# Full-Genome Sequence of Chicken Anemia Virus Strain GXC060821, Isolated from a Guangxi Sanhuang Chicken

**DOI:** 10.1128/genomeA.00040-14

**Published:** 2014-02-20

**Authors:** Zhixun Xie, Xianwen Deng, Liji Xie, Jiabo Liu, Yaoshan Pang, Zhiqin Xie, Qing Fan, Sisi Luo

**Affiliations:** Guangxi Key Laboratory of Animal Vaccines and Diagnostics, Guangxi Veterinary Research Institute, Nanning, Guangxi Province, China

## Abstract

We report here the complete genomic sequence of a novel chicken anemia virus strain GXC060821, isolated from a Sanhuang chicken in Guangxi Province of southern China. The complete genome of GXC060821 was sequenced. The full-length of GXC060821 is 2,292 bp and contains three overlapping open reading frames (ORFs). A comparison of the complete sequences and the deduced amino acid sequences of GXC060821 with 31 other published chicken anemia virus sequences showed that the homologies of the nucleotides are 96.1% to 98.5% and the homologies of the deduced amino acid sequences are 89.8% to 94.2%. Phylogenetic tree analysis indicated that GXC060821 is closely related to the two Chinese strains, TJBD40 (accession no. AY843527) and LF4 (accession no. AY839944), and it has a distant relationship with the American isolate 98D06073 (accession no. AF311900). This report will help to understand the epidemiology and molecular characteristics of chicken anemia virus in a Guangxi Sanhuang chicken.

## GENOME ANNOUNCEMENT

Chicken anemia virus (CAV) is a small DNA virus with a circular, covalently linked, single negative-strand genome. It is the causative agent of chicken infectious anemia (CIA) and classified in the family *Circoviridae*, genus *Gyrovirus* (1). CAV is an economically important pathogen with a worldwide distribution. CAV was first isolated in 1979 in Japan and is the major agent responsible for a disease causing severe anemia and immunosuppression (2). The characteristic symptoms of the disease include aplasia of the bone marrow and the destruction of T-lymphoid tissue, which has been shown histopathologically after CAV infection (3, 4). Generally, CAV as the causative agent of chicken anemia disease affects 1-day-old chicks that lack maternal antibodies (5). Mortality rates as high as 55% and morbidity rates as high as 80% have been described when chicks are infected with CAV (6, 7).

In this study, a novel strain of CAV, named GXC060821, was first isolated from a Sanhuang chicken in Guangxi, southern China. The nucleotide sequences of this strain were amplified by PCR. The amplified products were purified and cloned into the pMD-18T vector (TaKaRa, Dalian, China) and then sequenced (Invitrogen, Guangzhou, China) (8). The sequences were assembled and manually edited to generate the final genome sequence.

Sequence analysis showed that the full-genome sequence of GXC060821 is 2,292 nucleotides and contains 3 overlapping open reading frames (ORFs), including viral protein 1 (VP1), viral protein 2 (VP2), and viral protein 3 (VP3) (7–9). The full lengths of these ORFs are 1,350, 651, and 366 nucleotides, respectively.

GXC060821 was compared with 31 CAV stains, four American strains, one Australian strain, one German strain, two Japanese strains, six Malaysian strains, 10 Chinese strains, and seven strains from the United Kingdom. The nucleotide sequence identities of the VP1, VP2, and VP3 genes between GXC060821 and 31 CAV strains are 94.4% to 98.6%, 98.6% to 99.5%, and 98.6% to 99.7%, respectively, and the amino acid sequence identities are 96.7% to 99.6%, 96.8% to 98.6%, and 96.7% to 99.2%, respectively.

Immunogenicity studies have shown that VP1 and VP2 are crucial components for the elicitation of host-produced virus neutralizing antibodies in chickens (10), and the amino acid sequence of GXC060821 with other 31 CAV strains is conservative. Therefore, VP1 and VP2 have previously been thought to be good candidates for use as immunogens when developing subunit vaccines or diagnostic kits (10, 11).

The complete sequences and the deduced amino acid sequences of GXC060821 with 31 other published CAV sequences showed that the homologies of the nucleotides are 96.1% to 98.5% and the homologies of the deduced amino acid sequences are 89.8% to 94.2%. Phylogenetic tree analysis indicated that GXC060821 is closely related to the two Chinese strains, TJBD40 (GenBank accession no. AY843527) and LF4 (GenBank accession no. AY839944), and it has a distant relationship with the American isolate 98D06073 (GenBank accession no. AF311900). This report will help to understand the epidemiology and molecular characteristics of CAV in a Guangxi Sanhuang chicken.

### Nucleotide sequence accession number.

The complete genomic sequence of GXC060821 was deposited in GenBank under the accession no. JX964755.
